# *SYTL4* May Serve as a New Predictive Biomarker for Survival and Trastuzumab Treatment Responsiveness in HER2-Positive Breast Cancer

**DOI:** 10.3390/ijms27104533

**Published:** 2026-05-18

**Authors:** Pawel Kordowitzki

**Affiliations:** 1 Department of Basic and Preclinical Sciences, Nicolaus Copernicus University, 87-100 Torun, Poland; p.kordowitzki@umk.pl; 2 Institute of Advanced Studies, Nicolaus Copernicus University, 87-100 Torun, Poland; 3 Department of Gynecology with Center for Oncological Surgery, Campus Virchow Klinikum, Charité—Universitätsmedizin Berlin, 13353 Berlin, Germany

**Keywords:** breast cancer, Trastuzumab, Sytl4, HER2 positivity, biomarker

## Abstract

Breast cancer has emerged as the preeminent global health crisis in oncology, currently standing as the most frequently diagnosed malignancy among women worldwide. Establishing novel predictive biomarkers is paramount to truly personalize treatment approaches, minimize unnecessary toxicity, and significantly improve long-term outcomes for patients with breast cancer. Breast cancer transcriptomic datasets were retrieved from the Gene Expression Omnibus and processed through standardized normalization procedures. Mutation-driven regulation of *SYTL4* expression, treatment response to trastuzumab, cancer hallmark enrichment, and survival associations were evaluated using established bioinformatic tools and enrichment analysis based on integrated cancer hallmark gene sets. Additionally, DNA methylation profiles were analyzed. Herein, it is shown that *SYTL4* mRNA expression is significantly (*p* = 2.01 × 10^−4^) diminished in breast cancer bearing *BRCA1* mutations, suggesting a mechanistic interplay between *BRCA1*-driven genomic instability and *SYTL4*-regulated signaling cascades. Kaplan–Meier survival analysis demonstrated that elevated *SYTL4* mRNA expression is significantly associated with improved overall survival in HER2-positive breast cancer patients (HR = 0.72; *p* = 0.034). Consistently, *SYTL4* expression was significantly higher in patients who responded to trastuzumab therapy, supporting its potential as a biomarker of therapeutic response. Epigenetic analysis further revealed significant differential DNA methylation of *SYTL4* between tumor and unaffected control tissues (*p* < 2.2 × 10^−16^), with region-specific hypomethylation in tumor regulatory regions. KEGG pathway and cancer hallmark enrichment analyses indicated that genes with prominent methylation changes are involved in cytokine signaling, growth factor pathways, and extracellular matrix remodeling, with the strongest associations observed for hallmarks related to genome instability, replicative immortality, resisting cell death, and metabolic reprogramming. In summary, we present that the gene SYTL4 is a prospective biomarker for survival and trastuzumab treatment responsiveness. Our observations posit that *SYTL4* expression may signify a biological milieu conducive to sustained HER2 reliance and amplified therapeutic vulnerability.

## 1. Introduction

Breast cancer has emerged as the preeminent global health crisis in oncology, currently standing as the most frequently diagnosed malignancy among women worldwide [1,2]. Current projections suggest that the global burden will intensify significantly, with new cases expected to rise by 38% and deaths by 68% by 2050, disproportionately affecting regions with lower human development indices [3,4]. The epidemiological profile of breast cancer is inextricably linked to the cessation of ovarian function during menopause. Research indicates a positive correlation between the age at menopause and cancer risk; specifically, Mendelian randomization studies have demonstrated that an older age at menopause significantly increases the risk of breast cancer due to a prolonged window of endogenous hormonal exposure [5,6]. In postmenopausal women, metabolic dysregulation, often manifesting as metabolic syndrome, becomes a critical driver of tumorigenesis [7,8]. Systems of obesity, hypertension, and diabetes are independently associated with a higher incidence of the disease, as adipose tissue becomes the primary site for estrogen synthesis after ovarian decline [9,10]. Furthermore, the management of menopausal symptoms through hormone therapy (MHT) further modulates this risk. Large-scale cohort studies indicate that prolonged use of MHT, particularly combined estrogen–progestogen formulations, is associated with a significantly elevated risk of invasive postmenopausal breast cancer subtypes [9,10]. In this regard, it is worth mentioning that human epidermal growth factor receptor 2-positive (HER2+) breast cancer, once characterized by aggressive tumor biology and poor prognosis, has witnessed a transformative shift in its clinical trajectory with the advent of HER2-targeted therapies. The introduction of trastuzumab and subsequent anti-HER2 agents has profoundly reshaped outcomes, making HER2 positivity a positive predictive biomarker rather than a marker of poor outcomes [11,12]. Despite these remarkable advancements, HER2+ breast cancer remains a heterogeneous disease, and predicting individual patient response to treatment, particularly to trastuzumab, and long-term survival probability continues to be a significant challenge [12].

While targeting the HER2 tyrosine kinase is the mainstay of many current treatment algorithms, elevated HER2 protein levels alone are often insufficient to guarantee durable responses [12]. A complex interplay of tumor cell-intrinsic features, such as HER2 downstream signaling, *ERBB2* gene amplification, luminal differentiation, estrogen receptor levels, and proliferation, alongside microenvironmental characteristics like immune infiltration and stroma activation, dictates treatment response and relapse risk [12]. Interestingly, although *BRCA1*-associated tumors are predominantly triple-negative, they occasionally exhibit HER2 amplification, with studies indicating a low prevalence of approximately 2.1% to 10% in BRCA1 carriers [13,14,15]. The coexistence of *BRCA1* mutations with HER2-positivity and the substantial biological heterogeneity across HER2+ tumors drive diverse resistance mechanisms and varied clinical outcomes, underscoring the urgent need for a more nuanced understanding to optimize therapeutic strategies.

Current treatment planning is largely modulated by tumor stage and response assessment following neoadjuvant therapies, often based on anatomic risk and overlooking the intricate biology of the disease [16]. Antibody–drug conjugates (ADCs) such as trastuzumab emtansine and trastuzumab deruxtecan have revolutionized HER2-directed breast cancer therapy by enabling targeted delivery of cytotoxic payloads to malignant cells [17]. The efficacy of these agents relies on receptor-mediated endocytosis followed by trafficking to lysosomes for proteolytic degradation and payload release [18,19]. In consequence, there is a critical need for biologically based biomarkers that can precisely identify patients most likely to derive durable benefit from HER2-targeted therapies, such as trastuzumab and pertuzumab, and those who may require treatment intensification or alternative strategies. In this regard, it is worth mentioning the gene *SYTL4* (synaptotagmin-like 4), which is implicated in tumorigenesis and cancer development. It is notably overexpressed in certain cancer tissues, including triple-negative breast cancer, where high expression is linked to poor outcomes, particularly with paclitaxel therapy [20]. Furthermore, *SYTL4* encodes a Rab27 effector that regulates vesicle transport and microtubule stability, which are critical components of the endocytic pathway [21]. While high *SYTL4* expression is linked to chemoresistance in triple-negative breast cancer by modulating microtubule-dependent transport, its potential to impact ADC lysosomal processing in HER2+ models may stem from its role in governing intracellular vesicle routing and the Rab-mediated machinery required for efficient drug delivery to the lysosome [21].

Establishing novel predictive biomarkers is paramount to truly personalize treatment approaches, minimize unnecessary toxicity, and significantly improve long-term outcomes for patients with breast cancer. Therefore, in this study, we focused on transcriptomic data, mutation status, treatment responsiveness, epigenetic data, HER2-positivity, cancer hallmarks, and survival probability, establishing the *SYTL4* gene as a pivotal regulator and a robust predictive biomarker for breast cancer patients, to tackle this global disease early.

## 2. Results

To evaluate the clinical and molecular relevance of *SYTL4* in breast cancer, we performed a series of integrative analyses examining its association with *BRCA1* mutation status, patient survival, Trastuzumab therapeutic response, epigenetic regulation, and cancer hallmark enrichment.

### 2.1. BRCA1 Mutation Status in Association with SYTL4 mRNA Expression

**Figure 1:**
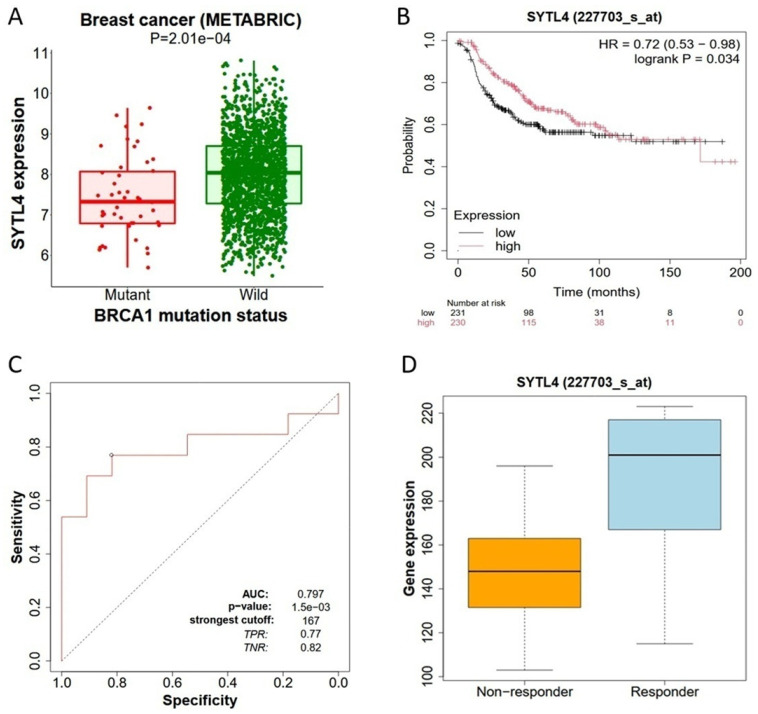
**SYTL4 mRNA expression, survival probability, and treatment responses.** Panel (**A**) shows SYTL4 messenger RNA expression in breast cancer samples from the METABRIC cohort (n = 1862), stratified by BRCA1 mutation status. Tumors harboring BRCA1 mutations (n = 46) exhibit significantly lower SYTL4 expression compared with wild-type (n = 1816) tumors (*p* = 2.01 × 10^−4^). The Mann–Whitney analysis was used for this panel. Panel (**B**) presents a Kaplan–Meier analysis of overall survival according to SYTL4 expression (high vs. low, dichotomized at the median) in HER2-positive breast cancer patients. High SYTL4 expression is associated with improved survival (hazard ratio for death, 0.72; 95% confidence interval [CI], 0.53–0.98; log-rank *p* = 0.034). Numbers at risk are shown below the plot. Panel (**C**) displays a receiver-operating characteristic (ROC) analysis comparing two cohorts, ‘treatment responders’ (n = 116) and ‘non-responders’ (n = 120), evaluating the performance of SYTL4 expression in predicting trastuzumab therapeutic response in HER2-positive breast cancer patients. The area under the curve (AUC) is 0.797 (*p* = 1.5 × 10^−3^), with a confidence interval of 95% (0.740–0.854). The optimal cutoff value (167) yields a true-positive rate (sensitivity) of 0.77 and a true-negative rate (specificity) of 0.82. The “strongest cutoff” value of 167 was determined using the Youden Index. Panel (**D**) illustrates SYTL4 expression levels according to trastuzumab treatment response status. Responders demonstrate significantly higher SYTL4 expression than non-responders, consistent with the survival and ROC analyses. Together, these findings support SYTL4 as a prognostic and predictive biomarker in breast cancer. The two cohorts (responders vs. non-responders) are compared using the Mann–Whitney test or the Receiver Operating Characteristic test in the R statistical environment. Statistical significance was set at *p* < 0.05.

Based on publicly available datasets (n = 1862 breast cancer samples encompassing over 25,000 genes) from the METABRIC breast cancer cohort, it was found that *SYTL4* mRNA expression was significantly lower in tumors carrying *BRCA1* mutations compared with *BRCA1* wild-type tumors (*p* = 2.01 × 10^−4^), and the median expression level was markedly higher in the *BRCA1* wild-type group, indicating that *BRCA1* mutational status is associated with altered *SYTL4* transcriptional regulation (Figure 1A). This finding suggests a potential link between SYTL4 expression and DNA repair–related tumor biology characteristic of *BRCA1*-driven breast cancer.

### 2.2. Prognostic Significance and Trastuzumab Treatment Response of SYTL4 in Regard to HER2-Positivity

Our Kaplan–Meier survival analysis demonstrated that *SYTL4* mRNA expression is significantly associated with survival in HER2-positive breast cancer patients. Patients with high *SYTL4* expression had improved overall survival compared with those with low expression. The survival curves diverged early and remained separated for more than 100 months of follow-up, with a hazard ratio (HR) of 0.72 (95% CI: 0.53–0.98) and a log-rank *p* value of 0.034 (Figure 1B), indicating that elevated *SYTL4* expression confers a protective prognostic effect. These results suggest that *SYTL4* may function as a favorable prognostic biomarker in breast cancer.

Next, we investigated whether *SYTL4* expression correlates with therapeutic responsiveness. Comparative analysis between treatment responders and non-responders revealed that *SYTL4* expression was significantly higher in HER2-positive breast cancer patients who responded to Trastuzumab treatment (Figure 1C). The responder group exhibited higher median expression levels and a broader distribution toward higher values, whereas non-responders showed lower overall expression levels (Figure 1D). These findings suggest that increased *SYTL4* expression is associated with improved treatment sensitivity and may serve as a predictive biomarker of therapeutic response. To further assess the discriminative potential of *SYTL4* expression, receiver operating characteristic (ROC) curve analysis was performed. The ROC analysis yielded an area under the curve (AUC) of 0.797, indicating good diagnostic accuracy (Figure 1D). The optimal cut-off value was 167, yielding a true positive rate (sensitivity) of 0.77 and a true negative rate (specificity) of 0.82. The statistical significance of the model was supported by a *p*-value of 1.5 × 10^−3^. These findings demonstrate that *SYTL4* expression has substantial discriminatory capacity and may serve as a useful biomarker for distinguishing clinically relevant breast cancer phenotypes.

### 2.3. Differential DNA Methylation of SYTL4 in Breast Cancer

**Figure 2:**
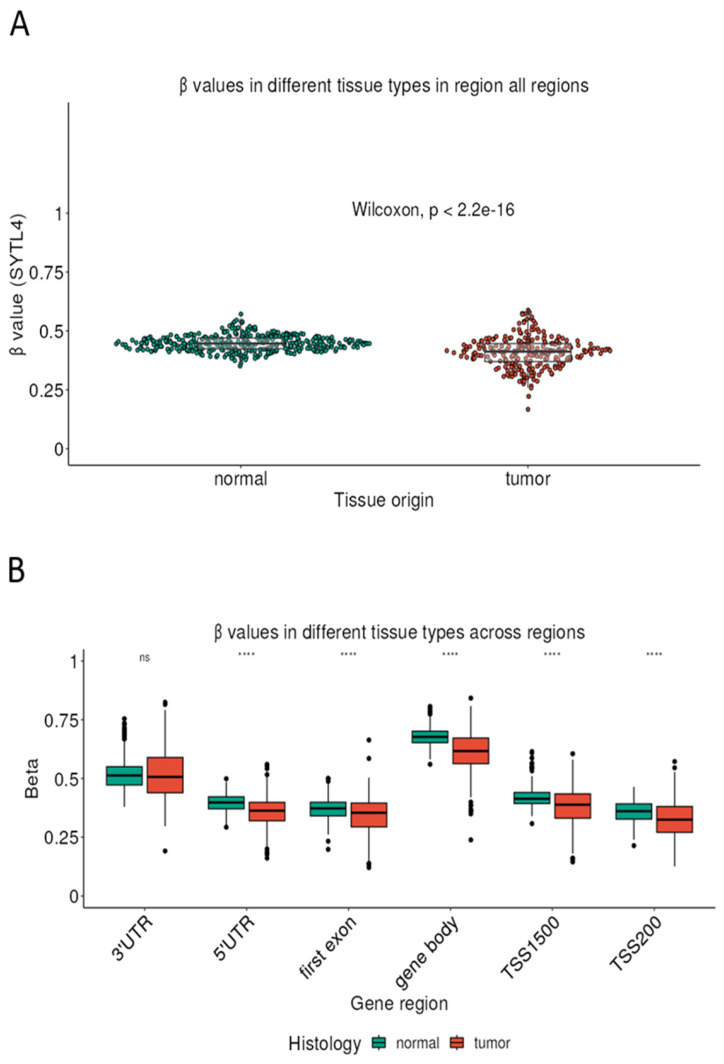
**DNA methylation status in breast cancer and control tissue**. Panel (**A**) shows overall DNA methylation levels (β values) of SYTL4 across all annotated genomic regions, comparing normal and tumor (breast cancer) tissues. Each dot represents an individual sample. Tumor tissues demonstrate significantly lower overall methylation compared to normal tissues (Wilcoxon test, *p* < 2.2 × 10^−16^). Panel (**B**) presents region-specific methylation levels stratified by gene annotation (3′ untranslated region [UTR], 5′ UTR, first exon, gene body, TSS1500, and TSS200) in normal (green) and tumor (red) tissues. Box plots depict median values and interquartile ranges, with whiskers indicating variability. Significant differences between normal and tumor tissues are observed across most regions (**** indicating *p* < 0.0001), except for the 3′ UTR (ns, not significant). Tumor samples show consistently lower methylation in promoter-associated regions (TSS1500, TSS200, 5′ UTR, first exon) and in the gene body.

To explore potential epigenetic mechanisms regulating *SYTL4* expression, we compared DNA methylation levels (β values) between breast cancer tissues and unaffected controls. The analysis revealed a significant difference in methylation levels, with tumors exhibiting altered methylation patterns compared with unaffected tissues. The difference was highly significant (*p* < 2.2 × 10^−16^), indicating robust epigenetic dysregulation of the *SYTL4* locus in breast cancer (Figure 2A). These findings suggest that DNA methylation may contribute to the transcriptional modulation of *SYTL4* during tumorigenesis. Further examination of methylation patterns across specific genomic regions of the *SYTL4* gene revealed region-specific alterations between unaffected and tumor tissues. Significant differences in β values were observed across multiple regulatory regions (Figure 2B), including the 3′ untranslated region (3′UTR), 5′UTR, first exon, gene body, and promoter-proximal regions (TSS1500 and TSS200). Tumor tissues displayed lower methylation levels in regulatory regions compared with unaffected tissues, suggesting widespread epigenetic remodeling across the *SYTL4* gene locus in breast cancer. These methylation changes may influence gene transcription and contribute to the observed differences in *SYTL4* expression.

### 2.4. KEGG Pathway Methylation and Cancer Hallmark Enrichment Analysis

**Figure 3:**
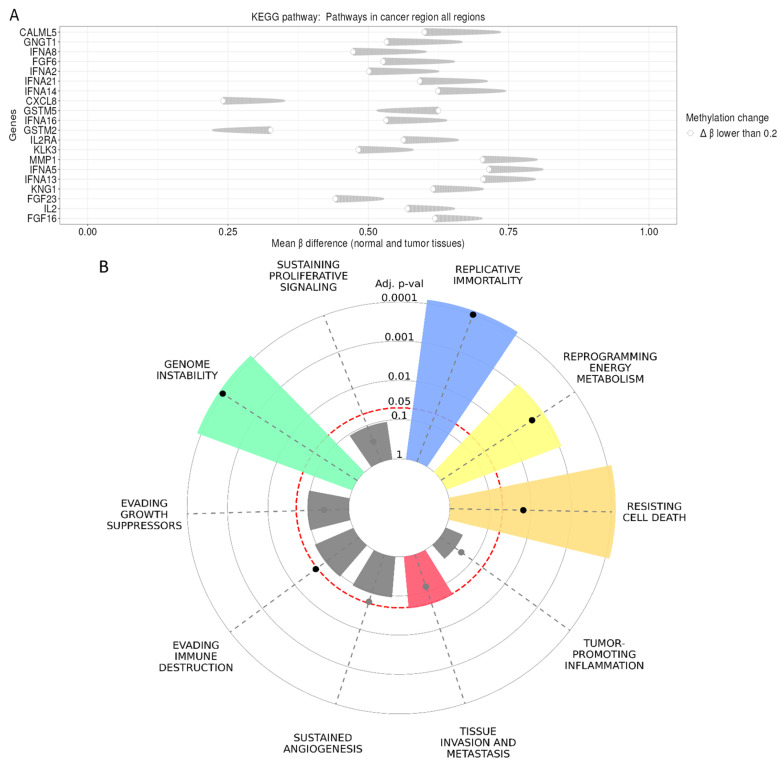
**KEGG Pathway cancer hallmark analysis.** Panel (**A**) depicts genes involved in the KEGG Pathways in breast cancer, showing significant differences in DNA methylation (β values) between normal (unaffected) and tumor tissues (breast cancer). Panel (**B**) presents a radial “hallmark fingerprint” summarizing enrichment of different cancer hallmarks for known breast cancer marker genes. The ten canonical hallmarks of cancer are visualized as discrete segments, with color applied only to those that reach statistical significance (*p* < 0.05). The proportional area of each segment denotes the magnitude of enrichment relative to the designated reference hallmark gene set. To optimize graphical resolution and visual clarity, the output axes are dynamically scaled according to the minimum *p*-value identified within the enrichment analysis. Gene regions were compared using the Kruskal–Wallis test, with pairwise post hoc comparisons using the Mann–Whitney test and a Bonferroni correction. The differentially methylated genes were analyzed in distinct regions with a ∆β value > 0.2, reflecting a change of ≥20% in methylation, which is considered functionally meaningful and a commonly accepted standard. Positive Δβ values indicate higher methylation in tumor tissue compared to normal tissue (hypermethylation), whereas negative Δβ values indicate lower methylation in tumor tissue compared to normal tissue (hypomethylation). The Abbreviation “p-val” stands for “*p*-value”.

The KEGG pathway methylation analysis showed that those genes with the most prominent methylation differences, in other words, pronounced shifts in mean β values, were IFNA family members (*IFNA1*, *IFNA2*, *IFNA13*, *IFNA14*, *IFNA16*), FGF family genes (*FGF6*, *FGF16*, *FGF23*), *CXCL8*, *IL2*, *IL2RA*, *MMP1*, *KLK3*, *GSTMS*, and *CALML5* (Figure 3A). These genes exhibited moderate to high methylation differences (mean β difference ~0.4–0.8), suggesting substantial epigenetic remodeling in tumor tissues. These patterns highlight a coordinated methylation shift affecting cytokine signaling, extracellular matrix remodeling, and growth factor pathways, all of which are known to contribute to tumor initiation and progression. To further assess the biological relevance of the identified gene set, we mapped the genes to established cancer hallmarks. The radial enrichment plot demonstrates that specific hallmarks are significantly overrepresented (Figure 3B). The strongest enrichment signals were observed for replicative immortality, genome instability, resistance to cell death, and reprogramming of energy metabolism, each with highly significant adjusted *p*-values (*p* < 0.001). Moderate enrichment was detected for tissue invasion and metastasis, whereas other hallmarks, such as sustained angiogenesis, evading immune destruction, and evading growth suppressors, showed weaker associations. These results indicate that the altered genes are primarily involved in fundamental mechanisms governing genomic integrity, cellular survival, and metabolic adaptation during tumor progression.

The quantitative enrichment analysis confirmed these findings (Table 1). The hallmark genome instability demonstrated the strongest association, with an overlap of 7/747 genes, an extremely significant *p*-value < 1 × 10^−6^, and an odds ratio of 96.24. Similarly, replicative immortality showed strong enrichment (5/547 genes, *p* = 0, odds ratio = 41.86), while resistance to cell death also showed a highly significant signal (7/1941 genes, *p* = 0, odds ratio = 33.75). Significant enrichment was additionally observed for reprogramming energy metabolism (4/740 genes, *p* = 0.00035, odds ratio = 19.36) and tissue invasion and metastasis (4/2318 genes, *p* = 0.02363, odds ratio = 5.48). The enriched gene sets included several key tumor suppressors and genome stability regulators, most prominently *TP53*, *PTEN*, *BRCA1*, *BRCA2*, *CHEK2*, *PALB2*, *STK11*, and *RIF1*, which are well-known mediators of DNA repair, cell cycle control, and apoptosis. Additional genes associated with metabolic regulation and tumor progression further contributed to the observed hallmark signatures.

## 3. Discussion

Our findings suggest, to the best of our knowledge, for the first time, the gene *SYTL4* as a potential and robust biomarker in breast cancer patients. Our study revealed a complex regulatory axis involving *BRCA1-mutation-mediated* expression and tumor-specific DNA hypomethylation, which collectively influence survival outcomes and therapeutic responsiveness to trastuzumab. The significant (*p* = 2.01 × 10^−4^) downregulation of *SYTL4* in *BRCA1*-mutated tumors, which were investigated by using the muTarget analysis tool, suggests that *BRCA1* may act as a positive regulator of *SYTL4* transcription, or that its deficiency leads to a cellular state in which *SYTL4* is actively repressed. The observed lower *SYTL4* mRNA expression in *BRCA1*-mutated tumors indicates a potential association with DNA repair–deficient tumor contexts, but this relationship should be interpreted cautiously. Rather than implying a direct regulatory interaction, this study supports the notion that *SYTL4* expression may reflect broader transcriptional or epigenetic changes occurring in *BRCA1*-driven tumors. Whether *SYTL4* plays an active functional role in these processes or serves as a downstream marker of altered cellular states remains to be further validated experimentally. Given the central role of *BRCA1* in maintaining genomic integrity and regulating various metabolic and secretory pathways, this link appears biologically plausible [22]. Notably, while high *SYTL4* expression has been associated with poor prognosis and taxane resistance in triple-negative breast cancer and acute myeloid leukemia, the present results demonstrate that high *SYTL4* levels correlate with significantly improved overall survival in HER2-positive patients, with a hazard ratio (HR) for death of 0.72 (*p* = 0.034) [23]. This discrepancy highlights the context-dependent role of *SYTL4*, potentially acting through different Rab-effector mechanisms or secretory pathways depending on the oncogenic drivers present in the tumor microenvironment [24].

Another interesting result of this study is the high predictive accuracy of *SYTL4* for trastuzumab treatment response, evidenced by an AUC of 0.797 (*p* = 1.5 × 10^−3^). Treatment responders exhibited significantly higher *SYTL4* expression, suggesting that *SYTL4* may facilitate trafficking or stability of the HER2 receptor or participate in the exocytosis of factors that sensitize cells to antibody-dependent cellular cytotoxicity. Since *SYTL4* is a known Rab27 effector involved in vesicle transport, its presence might be necessary for the efficient presentation of HER2 on the cell surface or the secretion of immune-modulatory proteins that enhance trastuzumab’s efficacy [24]. This observation provides a compelling rationale for screening HER2-positive patients for *SYTL4* expression to identify those most likely to benefit from trastuzumab, thereby refining precision medicine strategies. However, given the observational nature of this study and the lack of adjustment for key clinical confounders, these findings should be considered hypothesis-generating and stimulating new research projects, rather than definitive evidence of a causal relationship.

Epigenetic profiling revealed that *SYTL4* is overall significantly (*p* < 2.2 × 10^−16^) hypomethylated in tumor tissues compared with unaffected control tissues, particularly in promoter-associated regions and the gene body. This hypomethylation typically corresponds to increased transcriptional potential, which aligns with the observed overexpression of *SYTL4* in many cancer types compared to normal controls [25]. Nevertheless, the relationship between methylation and gene expression is complex and context-dependent; therefore, these findings should be interpreted as indicative of potential regulatory mechanisms rather than direct causal effects. In our KEGG pathway analysis, other genes, including *IFNA* family members, *CXCL8*, *KLK3*, *MMP1*, *FGF* genes, and *IL2RA*, exhibit significant mean β differences, predominantly reflecting tumor-associated hypomethylation (Δβ < 0.2). These results indicate consistent epigenetic deregulation of cancer-relevant genes in breast cancer compared with unaffected controls, suggesting altered transcriptional potential within oncogenic pathways. While DNA methylation changes are often associated with transcriptional regulation, we acknowledge that functional consequences cannot be inferred solely from methylation data.

Furthermore, the cancer hallmark fingerprint analysis of known breast cancer marker genes indicates that the aforementioned DNA methylation changes converge on core tumor programs, including replicative immortality, genome instability, and metabolic reprogramming. The significantly (*p* < 1 × 10^−6^) high odds ratio for genome instability (OR = 96.24) and the recurrent involvement of genes such as *BRCA1*, *BRCA2*, *CHEK2*, *PTEN*, *PALB2*, and *TP53* underscore the cooperative relationship between genetic mutations and epigenetic deregulation. The fact that *BRCA1* deficiency is associated with both *SYTL4* downregulation and broader epigenetic shifts suggests that loss of genomic integrity triggers a distinct “epigenetic fingerprint” that characterizes aggressive breast cancer phenotypes. The convergence of these alterations on metabolic adaptation further supports the idea that *SYTL4* and its regulatory network are integral to the tumor’s ability to navigate metabolic stress and evade cell death [26]. However, we emphasize that the enrichment results reflect associations at the pathway level and do not establish direct mechanistic interactions with *SYTL4*.

## 4. Methods

### 4.1. Datasets

Breast cancer datasets were identified within the Gene Expression Omnibus repository through the utilization of platform identifiers GPL96 (HG-U133A), GPL570 (HG-U133 Plus 2.0), and GPL571 (HG-U133A_2), alongside the search terms “breast,” “cancer,” and “therapy”, as recently published [27]. Datasets comprising fewer than 30 samples were excluded from the analysis to maintain statistical power, although several cohorts were initially larger before being reduced to relevant patient specimens. These platforms were prioritized for their widespread use in the field and for using identical probe sequences for gene measurement. When genes were represented by multiple probes, the Jetset19 tool was used to select the most reliable probe set. Raw CEL files were normalized using the MAS5 algorithm in the R statistical environment with the Affy Bioconductor library. Subsequently, a secondary scaling normalization was performed to set the mean expression of 22,277 identical probe sets in each array to 1000; this step was implemented to minimize batch effects arising from varying mean targets during normalization of the three human genome arrays [27]. In this study, results were generated using the METABRIC cohort (n = 1862) [28], including breast cancer patients at all stages, both ER-positive and negative tumors, unless specifically stated in the respective analysis, such as the HER2-positivity analysis.

### 4.2. Identification of Mutations Influencing the Expression of SYTL4

The muTarget cancer biomarker tool has been used to analyze how mRNA expression of *SYLT4* is affected by mutations that alter its expression. The analysis was run as previously described and established by Nagy and Gyorffy [28]. Briefly, algorithm development and data processing were executed with R v3.5.2. Somatic mutation data identified via Mutect2 and RNA-seq-based gene expression profiles were retrieved from „The Cancer Genome Atlas” repository. Summarization was performed using the MAFtools R Bioconductor package. For the analysis of cancer-specific somatic mutations, mutation annotation format files were utilized to facilitate the exclusion of low-quality signals and variants likely representing germline alterations. Mutation filtering was based on Mutect2 classifications, supplemented by additional hard filters requiring a minimum of 20× coverage and the presence of the alteration in at least five reads.

### 4.3. Prediction of Trastuzumab Treatment Response

To evaluate the predictive accuracy of *SYTL4* upon the trastuzumab treatment, the ROC plotter tool was used, as previously reported [27]. Briefly, patients were stratified into two cohorts, treatment responders and non-responders, based on clinical characteristics and pathological response criteria. For those undergoing neoadjuvant chemotherapy, a binary classification system was adopted in place of the traditional four-tier categories; patients with no residual histological evidence of tumor were defined as responders, while those with residual tumor tissue were categorized as non-responders. Patients receiving adjuvant therapy were stratified by survival status at five-year follow-up. In this analysis, gene expression profiles of patients who relapsed within five years were compared with those of patients who survived beyond the five-year threshold, with individuals censored prior to this point excluded from the study. Statistical comparisons between the cohorts were conducted using Mann–Whitney tests or Receiver Operating Characteristic analyses in the R statistical environment, employing Bioconductor libraries.

### 4.4. Cancer Hallmark Genes

Cancer hallmark genes have been analyzed by merging 6763 genes from available mapping resources on the CancerHallmarks online platform, as recently published by Menyhart et al. [29]. Briefly, the foundation for the enrichment analysis of hallmark genes was established through the integrated cancer hallmark gene set. Within this overrepresentation analysis, biological features, specifically genes, are evaluated to determine if their prevalence within a dataset significantly exceeds random expectation. A hypergeometric test is performed using the “enrich” function in the GSEApy Python package. Through this process, the gene frequencies in the provided dataset are compared with those in the reference set, enabling the identification of significant overrepresentation across the ten integrated hallmarks. The associated online platform was developed using the Flask Python web framework and is hosted on an Ubuntu web server. To ensure the validity of the enrichment analysis, input gene lists are filtered to include only those symbols approved by the Human Genome Organization Gene Nomenclature Committee.

### 4.5. Kaplan–Meier Survival Probability Analysis

The gene-specific Kaplan–Meier analysis was performed using the recently published online plotter tool [29]. Briefly, survival analysis was performed using the lifelines (v.0.27.4) package in Python v.3.10. Inclusion in the analysis was restricted to genes associated with at least 5 relapse events in at least one of the cohorts. Survival outcomes were characterized using Cox proportional hazards models and Log-Rank tests to evaluate prognostic significance.

### 4.6. DNA Methylation-Based Biomarker Analysis

The DNA methylation status analysis was performed using the EpigenPlot tool, an interactive web platform for DNA methylation [30]. EpignePlot was developed based on raw data files that were preprocessed within the R statistical environment utilizing the minfi (RRID:SCR_012830) and wateRmelon (RRID:SCR_001296) packages. Beta values were determined via the equation beta = M/(M + U + 100), where M represents the methylated signal intensity and U denotes the unmethylated signal intensity. Subsequently, type II probe bias was mitigated by implementing the beta-mixture quantile normalization method. Probes and samples were subjected to rigorous filtering based on detection *p*-values; specifically, probes and samples characterized by a detection *p*-value exceeding 0.01 in more than 80% of the dataset were excluded. Furthermore, cross-reactive, SNP-containing, and sex chromosome-located probes were eliminated from the analysis. For the investigation of specific gene regions, the HumanMethylation450 v1.2 and Infinium MethylationEPIC v1.0 manifest files were employed. Probes were stratified into six distinct categories based on UCSC RefGene groups—comprising TSS1500, TSS200, 5′UTR, first exon, gene body, and 3′UTR—and beta values were subsequently aggregated according to UCSC RefGene nomenclature.

### 4.7. Statistical Analysis

Gene functional annotation was performed using the BioMart R Bioconductor package to assign Gene Ontology categories, and the statistical significance of associations between differentially expressed genes and GO terms was determined via chi-square analysis. Inter-group differences were assessed using the Mann–Whitney test or Receiver Operating Characteristic analysis. The optimal diagnostic cutoff threshold for the ROC analysis was identified using the Youden Index. In the epigenetic analysis, global methylation levels across normal (unaffected) and breast cancer tissues were compared using the Wilcoxon test. Regional methylation disparities were evaluated using a Kruskal–Wallis test, followed by post hoc pairwise Mann–Whitney tests with Bonferroni correction. The differentially methylated genes were analyzed in distinct regions with a ∆β value > 0.2, reflecting a change of ≥20% in methylation, which is considered functionally meaningful and a commonly accepted standard. Positive Δβ values indicate higher methylation in tumor tissue compared to normal tissue (hypermethylation), whereas negative Δβ values indicate lower methylation in tumor tissue compared to normal tissue (hypomethylation). The specific statistical tests are mentioned in the respective figure legends.

## 5. Conclusions

In conclusion, *SYTL4* emerges as a multi-modal biomarker whose expression and methylation status provide critical insights into breast cancer biology. Its role as a positive prognostic and predictive factor in HER2-positive breast cancer demands further investigation into the subtype-specific molecular interactions of synaptotagmin-like proteins. Screening for *SYTL4* could significantly enhance the clinical management of HER2-positive patients by predicting trastuzumab sensitivity and long-term survival probability. Amid the prognostic heterogeneity in HER2-positive breast cancer, novel biomarkers to sharpen Trastuzumab response prediction are imperative. We thus endorse prospective appraisal of *SYTL4* mRNA expression as a potential clinical biomarker of response to HER2-directed therapies.

## 6. Limitations

Despite the significant associations identified, several limitations must be considered when interpreting these findings regarding *SYTL4*. First, the reliance on the METABRIC cohort and other publicly available datasets introduces the potential for cohort-specific biases. Without cross-cohort confirmation, the observed performance metrics may be overestimated. Furthermore, the survival outcomes were primarily evaluated using univariate Kaplan–Meier analysis. The lack of multivariable survival analyses adjusting for key clinical confounders, including tumor stage, estrogen receptor status, histological grade, and specific therapeutic regimens, precludes a definitive assessment of *SYTL4* as an independent prognostic factor. Future studies employing Cox proportional hazards models with comprehensive covariate adjustment and experimental validation of the proposed epigenetic mechanisms are essential to establish *SYTL4* as a clinically actionable biomarker in breast cancer. Moreover, future studies in a larger cohort to validate *SYTL4* as an adequate clinical biomarker should include RNA sequencing data from preoperative patients receiving HER2-directed therapy.

## Figures and Tables

**Table 1:** Table quantifying the overlap between the breast cancer marker genes and established hallmark gene sets.

Cancer Hallmark	Overlap	*p*-Value	Odds Ratio	Genes
Sustaining proliferative signaling	4/3574	0.09816	3.2	*PTEN*; *TP53*; *BRCA1*; *STK11*
Genome instability	7/747	<1 × 10^−6^	96.24	*CHEK2*; *BRCA2*; *BRCA1*; *PTEN*; *PALB2*; *TP53*; *RIF1*
Evading growth suppressors	4/3288	0.07555	3.56	*PTEN*; *TP53*; *BRCA1*; *STK11*
Evading immune destruction	2/749	0.05706	7.33	*PTEN*; *TP53*
Sustained angiogenesis	2/796	0.06364	6.87	*TP53*; *BRCA1*
Tissue invasion and metastasis	4/2318	0.02363	5.48	*PTEN*; *TP53*; *BRCA1*; *STK11*
Tumor-promoting inflammation	1/769	0.34369	3.7	*TP53*
Resisting cell death	7/1941	0.0	33.75	*CHEK2*; *BRCA2*; *BRCA1*; *STK11*; *PTEN*; *PALB2*; *TP53*
Reprogramming energy metabolism	4/740	0.00035	19.36	*PTEN*; *TP53*; *BRCA1*; *STK11*
Replicative immortality	5/547	0.0	41.86	*CHEK2*; *BRCA2*; *PTEN*; *TP53*; *RIF1*

## Data Availability

All datasets and online tools are publicly available and are mentioned in the respective Section 4.

## Abbreviations

The following abbreviations are used in this manuscript:

AUC	area under the curve
HER2	Human Epidermal Growth Factor Receptor 2
ROC	receiver operating characteristic
SYTL4	Synaptotagmin-like protein 4
